# Multi-scale in vivo imaging of tumour development using a germline conditional triple-reporter system

**DOI:** 10.21203/rs.3.rs-4196140/v1

**Published:** 2024-04-03

**Authors:** Piotr Dzien, Ximena Raffo Iraolagoitia, Stephanie May, David Stevenson, Lynn McGarry, Dmitry Soloviev, Gavin Brown, Colin Nixon, Chrysa Kapeni, Maike De La Roche, Karen Blyth, Scott Lyons, Thomas Bird, Douglas Strathdee, Gilbert Fruhwirth, Leo Carlin, David Lewis

**Affiliations:** Cancer Research UK Scotland Institute; Cancer Research UK Scotland Institute; Cancer Research UK Scotland Institute; Cancer Research UK Scotland Institute; Cancer Research UK Scotland Institute; Cancer Research UK Scotland Institute; Cancer Research UK Scotland Institute; Cancer Research UK Scotland Institute; Cancer Research UK Cambridge Institute; Cancer Research UK Cambridge Institute; CRUK Scotland Institute; Cold Spring Harbor Laboratory; Cancer Research UK Scotland Institute; Cancer Research UK Scotland Institute; Kings College London; Cancer Research UK Scotland Institute; Cancer Research UK Scotland Institute

## Abstract

Imaging reporter genes are indispensable for visualising biological processes in living subjects, particularly in cancer research where they have been used to observe tumour development, cancer cell dissemination, and treatment response. Engineering reporter genes into the germline frequently involves single imaging modality reporters operating over limited spatial scales. To address these limitations, we developed an inducible triple-reporter mouse model (Rosa26^LSL – NRL^) that integrates reporters for complementary imaging modalities, flfluorescence, bioluminescence and positron emission tomography (PET), along with inducible Cre-lox functionality for precise spatiotemporal control of reporter expression. We demonstrated robust reporter inducibility across various tissues in the Rosa26^LSL – NRL^ mouse, facilitating effective tracking and characterisation of tumours in liver and lung cancer mouse models. We precisely pinpointed tumour location using multimodal whole-body imaging which guided *in situ* lung microscopy to visualise cell-cell interactions within the tumour microenvironment. The triple-reporter system establishes a robust new platform technology for multi-scale investigation of biological processes within whole animals, enabling tissue-specific and sensitive cell tracking, spanning from the whole-body to cellular scales.

## Introduction

To develop a comprehensive understanding of complex diseases such as cancer, methods are needed that can correlate information between the cellular microscale and the whole-organism macroscale. Whole-body imaging provides valuable information on tumour growth, metastasis, and treatment response, but it lacks the resolution to image the cellular microenvironment. Conversely, microscale imaging can reveal dynamic interactions between cancer cells and their microenvironment, but without systemic context. A combined system would enable whole-body imaging to provide a contextual understanding of longitudinal tumour formation and use microscopy for a deeper analysis of the cellular context during significant events like metastatic seeding.

Imaging reporter genes encode proteins that produce a sensitive and specific signal for *in vivo* imaging [1]. The signal can mark clonally expanding transformed cells and track them *in vivo* throughout a tumour’s progression. This is particularly useful in the context of genetically engineered mouse models (GEMMs) of cancer [2]. These models develop lesions that typically develop at inaccessible locations with unpredictable latency. The need to monitor tumour development in these mouse models has led to the development of strategies that combine tumour initiation with the induction of reporter transgene expression. For conditional GEMMS, this is typically using the Cre-loxP system [3–6]. A number of reporter strains are widely utilised and are available for multiple modalities, working with bioluminescence [7, 8], flfluorescence [7, 9], or radionuclide imaging [10].

Conditional reporter systems can conceivably track tumour development from the time of tumour initiation. However, the imaging modalities capable of visualising events at the cellular level, such as intra-vital flfluorescence microscopy, have neither the penetrative tissue depth nor field-of-view to locate specific cell populations arising at most locations within the imaged subject [11, 12]. Conversely, the modalities that enable tomographic whole-animal imaging without penetration depth limitations, such as positron emission tomography (PET) or magnetic resonance imaging (MRI) do not have the spatial resolution for cellular imaging. These compromises make it unlikely that a single imaging modality can bridge the gap between macro- and microscopic imaging. Therefore, combination approaches potentially have huge value in marking a specific cell population, which can be followed at the whole-animal scale over time, whilst retaining the facility of zooming in to a microscopic process in the same cell population at a relevant time-point.

One way of achieving multiscale imaging is to engineer a fusion protein that comprises reporter modules covering the macro and micro imaging scales. Such a reporter would allow longitudinal monitoring of the target cell population with modalities such as PET or bioluminescence, and single cell detection, using intravital flfluorescence microscopy [13–15]. Imaging triple modality fusion reporter proteins has previously been demonstrated *in vivo*, but each reporter combination has required extensive optimisation and characterisation, indicating limited flexibility of this approach [13, 14]. To optimise reporter gene functionality and mitigate potential steric hindrance posed by a large fusion protein, we strategically inserted a 2A element between the NIS and luciferase transgenes. This approach is widely recognised to result in the equimolar expression of both transgenes from a single, shared promotor element [16]. It has also been shown that NIS function is minimally compromised with the addition of a fluorescent protein fused to its C-terminus [17]. By adopting that gene organisation, flfluorescence microscopy can be employed to visualise labelled cells, as well as the cellular localisation of expressed NIS reporter protein.

Here, we developed and characterised a multiscale imaging system, R26^LSL – NRL^. This system incorporates three distinct reporter genes: murine sodium iodide symporter (mNIS) [18] fused to TagRFP [17, 19], and separated from firefly luciferase (Luc2) [20] by a self-cleaving P2A sequence [16]. We enabled the temporal control and tissue specificity of reporter gene expression using the Cre-loxP system [5]. To validate the extent of Cre-dependent switching throughout the body, we crossed R26^LSL – NRL^ with strains that ubiquitously express Cre recombinase and observed robust induction of reporter gene expression across the majority of tissue types. We combined the R26^LSL – NRL^ allele with conditional GEMMs of liver or lung cancer to demonstrate that it can be effectively used to non-invasively monitor dynamic tumour burden by bioluminescence and PET imaging. Additionally, as a proof of concept, we used the R26^LSL – NRL^ mouse to show that whole-body PET-MRI and bioluminescence imaging can guide intra-vital flfluorescence microscopy to specific lung tumours *in vivo*. This enabled imaging of TagRFP expression at cellular resolution to visualise cell-cell interactions within the tumour microenvironment. This innovative approach has the potential to unveil a variety of biological important features including macroscopic growth kinetics and therapy response while simultaneously capturing microscopic vascular and immune cell dynamics. The R26^LSL – NRL^ reporter mouse establishes a novel platform technology for systemic multimodal and multiscale imaging, with visualisation spanning from the whole-body to cellular levels.

## Methods

### Materials

Cell culture media: DMEM (#11965092), RPMI1640 (#21870084), MEM Non-essential amino acids (#11140–035), 50 mM β-mercaptoethanol (#31350–010), Glutamax (#35050–061), L-glutamine (#25030081) and recombinant Leukemia Inhibitory Factor (LIF, #A35935) were purchased from ThermoFisher Scientific (Life Technologies). 2% Gelatin (#G1393–100ml), Gentamycin (# G1272–10ml) and foetal bovine serum (#F7524–500ml) were purchased from Merck Life Science UK (Sigma Aldrich). Puromycin (#ant-pr-1) was from Invivogen and G418-disulphate (#G4185) from Formedium.

### Radiopharmaceuticals

Saline solution of [^99m^Tc]TcO_4_^−^ (Tc-99m-pertechnetate) for intravenous injection was purchased from the West of Scotland Radionuclide Dispensary, NHS Greater Glasgow and Clyde, and diluted in cell culture media, for *in vitro*, as indicated, or in physiological saline solution for *in vivo* use, respectively. [^18^F]BF _4_^−^ (F-18-tetra uoroborate, TFB) sodium salt was produced in-house at the radiopharmaceutical unit of the West of Scotland Glasgow PET Centre and administered in physiological saline solution, as previously described [21].

### Bioluminescence imaging and substrate

IVIS Spectrum *in vivo* optical imaging system (PerkinElmer, Waltham, Massachusetts) was used with default acquisition settings (open emission filter, blocked excitation filter, medium binning, f stop 1), a FOV (field of view) of B, C or D (6.6, 13.3 or 22.8 cm respectively) and acquisition times between 0.5 and 60 s. D-Luciferin (XenoLight D-Luciferin - K^+^ Salt Bioluminescent Substrate, PerkinElmer, #122799) was made up as a 15 mg/mL filter-sterilised solution in PBS, pH 7.0. Images were acquired and analysed with LivingImage 4.5.4 software (PerkinElmer).

#### Generation and characterisation of R26^LSL – NRL^ embryonic stem (ES) cells and subsequent mouse lines.

HM1 mouse embryonic stem cells (mESC) [22] were grown on an irradiated DR4 MEF [23] monolayer in ES cell medium [DMEM, 15% FBS, 1x Glutamax, 1x NEAA (non-essential amino acids), 0.1 mM β-mercaptoethanol, 1000 U/ml LIF and 33 μg/mL gentamycin]. 8 × 10^6^ HM1 mESC were electroporated with 40ug of a SwaI-linearised Rosa26 targeting vector containing an expression cassette comprising the CAGSA promoter/splice acceptor, a lox-STOP-lox NeoR cassette and a compound cDNA encoding a mNIS-RFP fusion, P2A and Luciferase 2. Cells were plated onto DR4 MEF monolayers under ES cell medium supplemented with 200 μg/ml G418 for selection. Surviving colonies were picked onto 96-well plates and screened for correct targeting by PCR across both the 5’ and 3’ homology arms using internal primers from within the novel inserted sequence and external primers from genomic sequence outside the extent of the homology arms (CGCCTAAAGAAGAGGCTGTG and AGTGCAGTGGCACAGTCTTG for the 5’; ACGACCGCAGTTCCTATGAC and CACTGACCATCATGCCTCTG for the 3’).

Following identification of correctly targeted clones, mouse lines were generated by injection of ES cells into C57BL/6J mouse blastocysts according to standard protocols [24]. After breeding of chimeras, germline offspring were identified by coat colour and the presence of the modified allele was confirmed with primers specific for the optimised ROSA26-mNIS transgene (ATCCCCATCAAGCTGATCC and TACCACCCACGGTCGTGTAC; 659bp).

### Locus targeting analysis

For locus targeting analysis, two R26^LSL – NRL/wt^ mice were sacrificed and their spleen genomic DNA was extracted, processed according to Targeted Locus Amplification (TLA) protocol [25], sequenced and mapped at Cergentis B.V. (Utrecht, The Netherlands).

#### Functional in vitro analysis of mNIS and Luc2 activity

For functional *in vitro* analysis of mNIS and Luc2 activity and inducibility, the parent R26-lsl-mNIS-RFP-Luc2 cells were transfected by electroporation with pCAGGS-Cre-ires-PURO plasmid (courtesy of Prof Francis Stewart, University of Dresden). Cells were selected under puromycin (1 μg/ml) for two days and surviving cells re-plated (4.5×10^4^ or 9.0×10^4^ cells per plate) onto 10 cm dishes of irradiated MEFs. Individual clones were tested for Cre-recombination by PCR from sequences 5’ of the upstream loxP into the tripartite cDNA. Oligos used were: SAFor primer (GCTGTCTCATCATTTTGGCAAAG; mNISTagRFP INT Rev primer: TACCACCCACGGTCGTGTAC). Prior to functional assay experiments, the parent ES cells and a selection of clonal lines stably expressing the mNIS-RFP-Luc2 tripartite cDNA (henceforth called ‘PARENT’ and CRE1-CRE4, respectively) were weaned off the MEF feeder layer over three subsequent passages with progressively lower MEF monolayer density on plates coated with a solution of porcine gelatin in PBS (0.1% w/v).

#### In vitro bioluminescence assay experiments

Gelatin was used to coat 24-well, black, clear bottom plates (VisiPlate-24 Black, PerkinElmer). R26-mNIS-RFP-Luc2 cells described above were seeded in ES cell growth medium at 2.5 ×10^4^ cells per well and incubated in 5% CO_2_ at 37°C for approximately 48 h. For assay experiments, the medium was removed and 1 mL of fresh growth medium at 37°C and containing 150 μg/mL D-luciferin was added to each well. Plates were then transferred immediately to the IVIS Spectrum instrument and a single image using default settings was acquired 5 min after the addition D-luciferin-containing medium. Cells were then harvested by trypsinisation and cell counting was performed using CellDrop Automated Cell Counter (DeNovix). Total Flux [photons/s] measured for a region of interest (ROI) covering the whole of the well and normalised to cell number [photons/s/cell] was reported. Four wells were used as replicates, the experiment was conducted once.

#### In vitro radioactivity uptake experiments

R26-mNIS-RFP-Luc2 cells described above were seeded at 1×10^5^ cells per well in 12-well, gelatin-coated plates in ES cell medium and incubated in 5% CO_2_ at 37°C for approximately 48 h. For assay experiments, the medium was removed and 1 mL samples of medium containing, at application time, approximately 50 kBq/mL of ^99m^TcO _4_^−^, were added per well to plates containing the tested cells. At the end of incubations, 60 min from the addition of radioactivity, the medium was removed rapidly and the plates were washed immediately with 5 mL of ice-cold PBS per well. Cells were lysed by the addition of 1mL of RIPA Lysis and Extraction Buffer (Thermo sher Scientific, Life Technologies) per well, 10 min incubation on ice and 15 min gentle rocking at 4°C. Radioactivity of the lysates was measured using Hidex Automatic Gamma Counter (Hidex Oy, Turku, Finland). Events within the energy window of 15–200 keV were used in analysis as described previously [26]. Protein concentration assayed using Pierce^™^ BCA (bicinchoninic acid) Protein Assay Kit (ThermoFisher Scientific) was used for uptake normalisation. Four wells were used as replicates, the experiment was conducted once.

### Animal husbandry

All animal experiments were performed in accordance with UK Home Office licences PPL: PP4144283 (Lewis), PP0604995 (Bird), PP8993874 (Strathdee), and 70/8645 (Blyth) and in accordance with the UK Animal (Scientific Procedures) Act 1986 and EU direction 2010. They were subject to review by the animal welfare and ethical review board of the University of Glasgow. Mice were housed under controlled conditions (individually ventilated cages in a dedicated barrier facility, 12 h light-dark cycle, 19–22°C, 45–65% humidity) with access to food and water ad libitum, and environmental enrichment. No randomisation or blinding was performed, no mice were excluded unless otherwise indicated.

Alleles used in this study were: *Gt(ROSA)26Sor*^*tm23(CAG*–*LSL*–*mNIS*–*RFP*–*P2A*–*Luc2*– *INV)Bea*^ **(R26**^**LSL – NRL**^), *Ctnnb1*^*tm1Mmt*^ **(Ctnnb**^**lox(ex3)**^) [27], *Gt(ROSA)26Sor*^*DM – lsl–Myc*^ **(R26** ^**LSL–MYC**^) [28], *Kras*^*tm4Tyj*^ **(Kras**^**LSL–G12D**^) [29], *Trp53*^*tm1Brn*^ **(p53**^**fi**^) [30], *Tg(CMV-cre)1Cgn* **(CMV-cre)** [31], *Gt(ROSA)26Sor*^*tm2(cre/ERT2)Brn*^ **(R26**^**cre – ERT2**^) [32]. Mixed background mice of both sexes were used unless otherwise specified. Ear notches collected at 3–4 weeks of age for identification were used by Transnetyx (Cordova, TN, USA) for genotyping.

#### Abbreviations of mouse genotypes:

NRL: R26 ^LSL-NRL/wt^
NRL-CE: R26^LSL-NRL/Cre-ERT2^
NRL-CV: R26 ^wt/LSL-NRL^ + CMV-Cre
NRL-KP: R26^LSL-NRL/wt^ + Kras^LSL-G12D/wt^ + Trp53^fl/fl^
KP: Kras^LSL-G12D/wt^ + Trp53^fl/fl^
NRL-BM: Ctnnb1^ex3/wt^ + R26^LSL-MYC/LSL-NRL^
BM: Ctnnb1^ex3/wt^ + R26^LSL-MYC/wt^

#### Tumour induction

Liver tumours were induced in 8–10 week old, male NRL-BM or BM littermate control mice using AAV8.TBG.PI.Cre.rBG (AAV8-TBG-Cre; Addgene#107787-AAV8) viral vector (Adeno-associated virus 8 with payload under Thyroxin Binding Globulin promoter with hepatocyte tropism) [33]. Control NRL-BM animals were infected with AAV8.TBG.PI.Null.bGH virus (AAV8-TBG-Null; Addgene#105536-AAV8) serving as a genetic control. Virus (6.4×10^8^ genomic copies/mouse) was diluted in 100 μl PBS and injected via the tail vein [34]. Post induction bioluminescence imaging started from day 17 and continued approximately every two weeks until endpoint. PET-MR imaging was performed near endpoint

Lung tumours were induced in 12–14 week old male and female NRL-KPor KP littermate control mice by bilateral intranasal inhalation of 5 × 10^7^ plaque forming units (PFU) of Ad5CMVCre as CaCl_2_ (9.5mM) precipitate in Modified Eagle’s Medium (Sigma #M-0268) prepared according to the producer’s (University of Iowa Viral Vector Core, Iowa City, USA) protocol [35] and administered in a total volume of 70 μL per mouse [36]. Post induction bioluminescence imaging started from day 30 and continued approximately every month until endpoint. PET-MR imaging was performed near endpoint.

#### Tamoxifen induction

Tamoxifen powder (Sigma #T5648) was dissolved with sonication in warm (37 °C) ethanol at 20 mg/mL. This was then dissolved at 1:9 in sunflower oil (Sigma #S5007) to a final concentration of 2 mg/mL tamoxifen. Unless stated otherwise, a total of four doses, each containing 20 mg tamoxifen per kg body weight, was administered i.p. at 48 h intervals to induce activation and nuclear translocation of cytoplasmic cre-ERT2. Further experiments were carried out between 7 and 21 days from the administration of the last dose of tamoxifen.

#### *In vivo* bioluminescence imaging (BLI)

Adult male and female mice were injected i.p. with 150 mg/kg D-Luciferin solution, anaesthetised with 2% isoflurane in 95% O_2_, transferred to the IVIS instrument, and a single image was acquired 20 min from D-Luciferin injection using default settings of stage-height D and exposure times between 0.5 s and 60 s.

#### *Ex vivo* bioluminescence imaging for R26-LSLmNIS-RFP-Luc2 induction

For measurements of organ bioluminescence, adult male and female mice were imaged as described for BLI, then culled by cervical dislocation immediately after imaging. Tissue samples were then rapidly collected and placed, one sample per well, in black, clear-bottom tissue culture plates (6-well plates, Corning^™^ Falcon^™^ or 24-well plates, PerkinElmer) using passive measures preventing temperature loss by the tissues (insulated dissection tables and transport boxes). The plates were then transferred into the IVIS instrument, where the tissue samples were equilibrated thermally for 2–3 min. A single image was then acquired 40 min from D-Luciferin injection using default settings with a FOV on stage height D and exposure times between 0.5 s and 60 s. Total flux [photons/s] was measured with an ROI covering the whole of the well and normalised to tissue weight and reported [photons/s/mg].

#### *In vivo* radioactivity biodistribution

Adult male and female mice were anaesthetised and intravenously injected with radiopharmaceutical ([^99m^Tc]pertechnetate or [^18^F]TFB, see below), as described for [^18^F]TFB PET-MR imaging experiments. After 90 minutes anaesthetised uptake animals were sacrificed and tissue samples were collected and weighed. Radioactivity from tissue samples was then measured using a Hidex Automatic Gamma Counter (Hidex Oy, Turku, Finland). Events within the energy window of 15–200 keV ([^99m^Tc]pertechnetate) or 400–800 keV ([^18^F]TFB) were used in analysis. Injected dose was calculated as the product of the expelled syringe volume and volumetric activity obtained from standard volume-activity curve of the injected stock, measured as described above and decay corrected to the same reference time. Standard uptake values were calculated as follows:

$$\text{SUV}=\frac{\text{c}\;\text{tissue}/\text{W}\;\text{tissue}}{ID/BW}$$

where c_tissue_ is tissue activity (Bq) ,W_tissue_ is tissue wet weight (g), ID is the injected dose (Bq), and BW is the body weight of the animal (g).

#### PET-MR imaging and data analysis

Adult male and female mice, anaesthetised with 1.0 – 2.5% isoflurane in 95% concentrated oxygen, were injected intravenously with 0.35–0.45 MBq of [^18^F]TFB per g body weight in 200–250 μL saline (0.9% NaCl) via a tail vein cannula and then transferred to a NanoScan PET/MRI (1T) (Mediso, Hungary). The respiration rate of the animals was monitored by pneumatic pad for the duration of the imaging session and their body temperature was maintained by flow of warm air. Coronal T1-weighted images, used for anatomical reference and attenuation correction, were acquired using 3D gradient-recalled echo sequence (TR 22.5 msec; TE 3.8 msec; flip angle 30°; data matrix, 256×256; slice thickness 0.70 mm; 48 slices). A 20 min static PET image was then acquired, starting 70 min from the injection of [^18^F]TFB, as described previously [26].

Images were reconstructed with Nucline software (Mediso, Hungary) using T1 3D GRE images for attenuation correction. PET/MR data were analysed using VivoQuantTM multi-modality post-processing suite (Invicro, USA). SUVmax was calculated with regions of interest over the whole of the tumour volume.

Maximum standardized uptake values (SUV) were calculated using:

$$\text{SUVmax}=\frac{\text{c}\;\text{img}}{ID/BW}$$

where c_img_ is the maximum activity concentration (MBq/mL) derived from the image ROI, ID is the injected dose (MBq), and BW is the body weight of the animal (g). One mL of tissue was assumed to weigh 1 g.

#### Flow Cytometry Quantification

Single cell suspensions of blood (by cardiac puncture) were collected from NRL-CV, NRL and wild-type adult male and female mice and stained for flow cytometry. Antibodies used: CD3-BV785 (17A2, BD Biosciences, cat 564010), CD45-BV711 (30-F11, Biolegend, cat 103147), CD4-PE-Cy7 (RM4–4, Biolegend, cat 116016), CD8-BUV395 (53–6.7, BD Bioscience, cat 563786), CD19-BV605 (6D5, Biolegend, cat 115540), NKp46-BUV737 (29A1.4, BD Bioscience, cat 612805), LY6G-APC-Cy7 (1A8, Biolegend, cat 127623), CD11b-BV650 (M1/70, Biolegend, cat 101259), SiglecF-AF647 (E50–2440, BD Bioscience, cat 562680), CD64-PerCPCy5.5 (X54–5/7.1, Biolegend, cat 139308), TCRγδ-FITC (GL3, Biolegend, cat 118105), CD11c-BV510 (N418, Biolegend, cat 117353) and counterstained with DAPI (ThermoFisher, cat D1306). Samples were acquired on a BD Fortessa instrument. Data were analysed using FlowJo v10.8.1. For cell gating, RFP+ cells were subgated out of CD45+DAPI− single and the immune subsets were subgated out of the RFP+ gate (see Table 1 and Supplementary Figure 4a).

#### Precision Cut Lung Slices (PCLS)

Mice were humanely killed by i.p. injection of sodium-pentobarbital and, after instillation of 1.1 mL of low-melting point agarose (2% in PBS w/v; Merck) through the trachea using a customized blunted 22G needle, lungs were excised en-bloc. Lungs were sliced into 300 μm thick sections on a vibrating microtome (Campden Ltd) either fixed in 4% formaldehyde in PBS (VWR) for 24h at 4°C, or fresh for immediate live imaging. Fixed slices were permeabilised and blocked in PBS with 0.3% Triton-X (Sigma), 10% normal goat serum (Sigma), 1% bovine serum albumin (Sigma), and 0.001% Sodium-Azide (VWR, UK); stained with hamster anti-CD31 (clone 2H8, Abcam UK), then anti-hamster AF488 (Jackson ImmunoResearch), anti-CD45 AF647 (Biolegend) and DAPI (Invitrogen). Finally, slices were mounted in Ce3D tissue clearing solution (Biolegend).

Whole tissue sections were imaged to a depth of 195 μm (66 planes) using an Opera Phenix high-throughput confocal microscope with 20x water objective (Perkin Elmer). Images were displayed as maximum projection of 66 planes separated by 3 μm, auto-contrasted and flat-field corrected. Analysis of stitched maximum projection images, was carried out using Harmony 4.9 image analysis software (Perkin Elmer). The analysis sequences were tailored to each tissue section. Tumours were segmented on the basis of high DAPI (Ex 405nm) intensity values and, where required, homogeneous DAPI texture. Tag-RFP-positive regions were defined as those where intensity (Ex 561nm) was above background values for the section. CD45-positive cells were segmented by intensity at 640nm.

Alternatively, fresh slices were stained with anti-CD31 AF647 (clone 390; Biolegend), anti-CD45 AF488 (clone 30-F11; Biolegend) and Hoechst33342 (Thermo Scientific, UK) in complete medium (phenol red-free DMEM supplemented with 1% FBS, Gibco) for 15 minutes at 37°C. Slices were imaged on a Zeiss LSM880 confocal microscope with Airyscan in a full incubation chamber at 37°C with 5% CO_2_. Airyscan processing of time-lapse sequences was performed in Zeiss Zen Software before visualisation with Imaris software (Bitplane, Oxford Instruments).

#### Simulated Lung Intravital Microscopy (S-LIVM)

Adult male and female mice injected intravenously with fluorescently conjugated mAb against CD31 (AF488) and CD45 (AF647) were humanely killed by i.p. injection of pentobarbital (Euthatal). After confirmation of death by severing the femoral artery, mice were mechanically ventilated (Physiosuite, Kent Scientific) with room air at 10 ml/g stroke volume and 150 breaths per minute with 0.1 cm/g positive end-expiratory pressure (PEEP). A custom-built flanged vacuum chamber with 8 mm glass cover slip, was inserted via a 5 mm incision between 2 ribs above the left lung lobe. The location had previously been identified using PET/MRI and bioluminescence imaging and was marked in black ink. Minimal suction (0.1–0.2 bar) was used to stabilise the lung against the coverslip. Imaging was performed on an upright LSM 880 NLO 2 photon / confocal microscope (Zeiss) using a 20× 1N.A. water immersion objective. Signals from the different fluorophores were captured simultaneously in three channels by optimising the emission wavelengths captured by PMT1 (AF488), GaAsP array (configured as a single detector 2; TagRFP) and PMT2 (AF647). 4D data were acquired by performing timelapse, z-stack, acquisitions over a volume with approx. 30 μm depth in 10 z-slices.

#### *In Situ* Hybridisation

Animals were sacrificed by CO_2_ inhalation unless stated otherwise. Tissues of interest were rapidly excised and fixed in 4% Neutral Buffered Formalin changed to 70% ethanol: 30% dH_2_O mixture after 24 hours, then paraffin embedded and stored at room temperature prior to sectioning. All *in situ* hybridisation (ISH) staining was performed on 4 μm formalin fixed paraffin embedded sections (FFPE) which had previously been incubated at 60 C for 2 h. ISH detection for, Mm-*Slc5a5* (487728), Mm-*Ppib* (313918) and *Dapβ* (312038) (Bio-Techne, USA) mRNA was performed using RNAScope 2.5 LSx (Brown) detection kit (322700; Bio-Techne, USA) on a Leica Bond Rx autostainer according to the manufacturer’s instructions. To complete the ISH staining, sections were rinsed in tap water, dehydrated through a graded ethanol series and placed in xylene. The stained sections were coverslipped in xylene using DPX mountant (SEA-1300–00A, CellPath).

#### ISH Image Analysis

Slides were scanned at ×20 magnification using a Leica Aperio AT2 instrument and analysed using the HALO image analysis platform (Indica Labs, Albuquerque, NM, US). HALO CytoNuclear v1.6 macro, tuned to identify negative (N) and weakly (W), moderately (M) or strongly (S) DAB-stained cells. H-score, defined below, was reported:

$$H-\text{Score}=\frac{2\text{S}+2\text{M}+1\text{W}}{\text{S}+\text{M}+\text{W}+\text{N}}\times 100$$

#### Statistical Analysis

Prism (GraphPad v10) was used to perform statistical analyses and to plot the data. In figures, error bars represent one standard deviation unless otherwise stated. P value classifications are summarised as follows: ns, p > 0.05; *, p < 0.05; **, p < 0.01; ***, p < 0.001.

## Results

### Rosa26 locus targeting and ES cell selection and characterisation.

**Figure 1:**
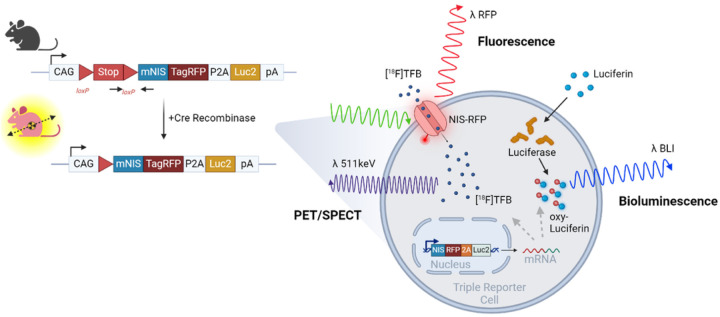
The triple-reporter gene mouse. Schematic of the transgene CAG-LSL-mNIS-RFP-P2A-Luc2 in the Rosa26 locus. Cre recombination deletes the lox-STOP-lox (LSL) sequence and switches on functional mNIS, TagRFP and Luc2 activities in the reporter cell of interest, which mediates corresponding [^18^F]tetrafluoroborate ([^18^F]TFB) PET, RFP fluorescence, and luciferin bioluminescence for multimodal imaging.

To generate an inducible germline triple-reporter system we targeted the Rosa26 locus in HM1 mouse embryonic stem cells with a transgene element CAG-LSL-mNIS-RFP-P2A-Luc2 allowing Cre-dependant expression of a tripartite cDNA comprising a fusion of mNIS *(Slc5a5)* and RFP alongside Luc2 (Fig. 1). To demonstrate the function of mNIS and Luc2 *in vitro* we performed radiolabelled pertechnetate ([^99m^Tc]TcO_4_^−^) uptake and bioluminescence assays on the Cre-deleted NRL-ES-Cre lines (CRE 1–4) and parent NRL-ES cells. We observed an 8.2 to 11.4-fold induction of [^99m^Tc]TcO_4_^−^ uptake and a 137 to 364-fold induction of bioluminescence in the NRL-ES-Cre cell lines compared to the parent NRL-ES line (Supplementary Fig. 1a and 1b). These measurements confirmed that the transgene reporter construct integrated into the parent ES cell line’s genome and was switched on by Cre-recombinase-mediated deletion of the lox-STOP-lox sequence in the NRL-ES-Cre cells. We verified the location and copy number of the transgene by performing targeted locus Amplification, sequencing, and data mapping, showing that a single copy of the vector integrated correctly at the targeted location (mouse chr6:113,076,032–113,076,035, an intron of Gt(ROSA)26Sor. There was no evidence of integration of genomic *E. coli* or vector backbone sequences.

### Crosses with strains ubiquitously expressing Cre recombinase

**Figure 2:**
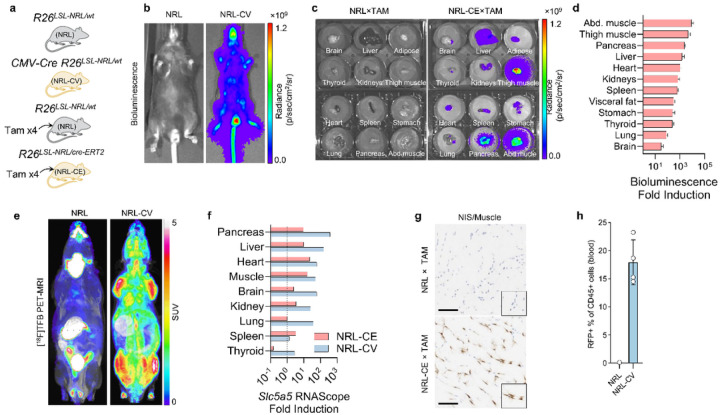
Multiscale imaging is enabled in composite mice combining NRL reporter and ubiquitous Cre alleles. **(a)** Mice heterozygous for R26^LSL-NRL^ were crossed together with mice carrying ubiquitous Cre either the constitutively active CMV-Cre (CV) or tamoxifen-inducible R26^Cre-ERT2^ (CE). Cre-recombination was spontaneous in composite NRL-CV offspring or induced by tamoxifen administration in NRL-CE offspring, respectively. **(b)** Example BLI images of NRL and littermate NRL-CV animals acquired 20 minutes after an i.p. injection of 15 mg/kg of D-Luciferin. **(c)** Example bioluminescence images of organs harvested from NRL (left) or littermate NRL-CE (right) mice that had been treated with four i.p. injections of tamoxifen at 20 mg/kg body weight. **(d)** Induction of Luc2 activity expression in NRL and littermate NRL-CE animals. Bioluminescence imaging of tissue samples harvested from NRL (n=4) or littermate NRL-CE (n=4) mice was performed 40 minutes after i.p. injection of D-Luciferin solution at 15 mg/kg body weight, and 20 minutes after sacrificing the mouse. Photon flux was normalised to sample weight (p/s/mg) and tissue type averages were calculated for each group. Mean arithmetic ratios with standard error of the mean (NRL-CE: NRL) are presented. See also Supplementary Figure 2a. **(e)** Example static [^18^F]TFB-PET MRI images of NRL and littermate NRL-CV animals acquired between 70–90 minutes after an i.v. injection of 0.5 GBq/kg of [^18^F]TFB. (f) Fold induction of Slc5a (mNIS) expression in NRL-CE and NRL-CV mice compared to littermate NRL controls as estimated by RNAScope detection of *Slc5a5* mRNA. Sections of tissue samples (n=1 mouse for each group) were used for RNAScope detection which was quantified using HALO analysis suite (positive stain H-score, Supplementary Fig. 3a and 3b). Arithmetic ratios (NRL-CV: NRL and NRL-CE: NRL) are presented. **(g)** Examples of *Slc5a5* RNAScope images of thigh muscle sections harvested from NRL or littermate NRL-CE mice. Scale bars are 100 μm. **(h)** Abundance of RFP-Tag-positive RFP immune (CD45+) cells in blood collected from NRL-CV (n=4) mice and a NRL littermate (n=1) animal. Error bars represents one standard deviation of the mean.

After validation of Cre-dependent reporter transgene function, we generated R26^LSL – NRL^ transgenic mice with the NRL-ES cells. To quantify induction efficiency of the reporter cassette in vivo across different tissue types, we crossed the R26^LSL – NRL^ mice with mice ubiquitously expressing Cre recombinase, either constitutively active (CMV-Cre [31]) or the tamoxifen-inducible Cre isoforms (R26^Cre − ERT2^ [32]). We placed NRL-CE (R26^LSL – NRL/Cre−ERT2^) mice and littermate NRL controls on a regimen of tamoxifen i.p. injections (Fig. 2a).

Whole-animal bioluminescence imaging showed high light output across the whole NRL-CV (R26 ^wt/LSL−NRL^ + CMV-Cre) mice relative to the littermate NRL controls, indicating robust induction of Luc2 activity expression (Fig. 2b). Following tamoxifen injection in NRL-CE animals and littermate NRL controls, we observed induction of Luc2 activity in all tissue types tested ranging from 31-fold (brain) to 8756-fold (abdominal muscle) (Fig. 2c, 2d and Supplementary Fig. 2a).

[^18^F]TFB PET-MRI imaging showed widespread mNIS-mediated tracer uptake particularly evident in skeletal muscle and the heart of the NRL-CV animals in comparison to the NRL controls (Fig. 2e). Similarly, technetium pertechnetate ([^99m^Tc]TcO _4_^−^) biodistribution indicated that average radioactivity uptake in skeletal muscle and heart of tamoxifen-treated NRL-CE animals was higher (ratio to control of 6.4 and 3.6-fold, respectively) when compared to NRL littermate controls (Supplementary Fig. 2b). For the remaining tissue types, average [^99m^Tc]TcO_4_^−^ uptakes appeared lower in NRL-CE animals (ratios to control in the range of 0.08–0.90). This was accompanied by radioactivity depletion from blood and urine (ratio to control of 0.06 and 0.10, respectively), consistent with rapid and avid uptake of the tracer by the large biomass of skeletal and cardiac muscle and the evidence of the ‘sink effect’ [37]. The absolute level of [^99m^Tc]TcO_4_^−^ uptake in the brain was low for both experimental groups (Supplementary Fig. 2b), which can be attributed to the blood-brain barrier restricting the passage of the tracer from blood into the brain’s extracellular space [38].

RNAScope detection of mNIS (*Slc5a5*) mRNA showed high levels of induction at the transcript level across most tissue types (Fig. 2f). The positive stain H-score ratio of NRL-CV to NRL mice was between 2.8-fold in the thyroid and 383.7-fold in the pancreas. NRL-CV spleen had an H-score similar (ratio of 1.33) to the NRL littermate control (Fig. 2f and Supplementary Fig. 3a). There were also high levels of mNIS induction across most tissue types following tamoxifen induction in NRL-CE mice, albeit slightly less than for NRL-CV (Fig. 2f and Supplementary Fig. 3b). Values ranged from 2.5-fold induction in the brain to 22.4-fold in the heart. The exception was the thyroid (ratio of 0.15) and lung (ratio of 1.02). The expression was uniform in the muscle (Fig. 2g), heart and pancreas but was heterogeneous in the brain, liver and spleen (Supplementary Fig. 3c). To demonstrate induction in immune cells, we performed flow cytometry on blood from NRL-CV mice, showing TagRFP expression in 17.9 ± 4.0% of CD45 + cells (Fig. 2h and Supplementary Fig. 4a). Interestingly, from each cell subset of total leukocytes, most were negative for TagRFP expression apart from neutrophils (49.41 ± 3.2%; Supplementary Fig. 4b). Taken together, these results show that a single copy of the R26^LSL – NRL^ allele is inherited in a Mendelian fashion and when the expression is switched on by Cre recombination this endows the carrier animals with functional expression of mNIS, TagRFP and Luc2 in a wide range of tissue types, facilitating multimodal imaging.

### In vivo multimodal imaging of oncogenesis in the Ctnnb1^ex3/wt^ R26^LSL − MYC/LSL−NRL^ (NRL-BM) GEMM of hepatocellular carcinoma.

**Figure 3:**
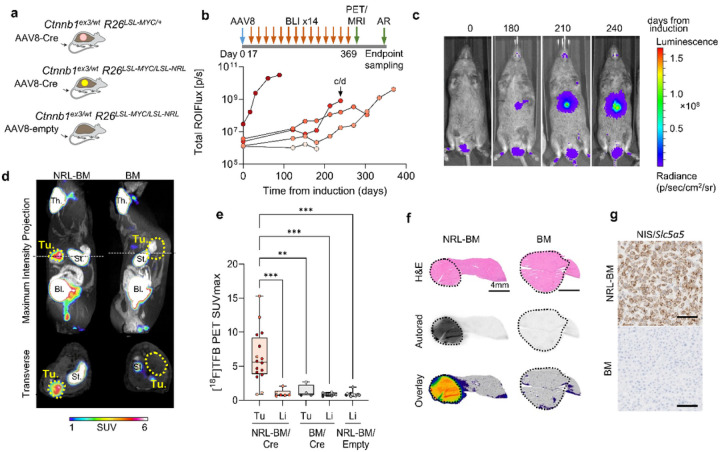
*In vivo* multimodal imaging of oncogenesis in the *Ctnnb1*^ex3/wt^ *R26*^LSL-MYC/LSL-NRL^ (NRL-BM) GEMM of hepatocellular carcinoma. **(a)** R26^LSL-NRL^ reporter mice were crossed with two gain-of-function Cre-dependent alleles *Ctnnb1*^ex3/wt^ and *R26*^LSL-MYC/wt^ (BM) to generate a conditional mouse model of hepatocellular carcinoma. Liver tumours were induced by intravenous injection of AAV8-TBG-Cre into NRL-BM and BM (littermate control) mice with AAV8-TBG-Empty used as a control vector. **(b)** Experiment timeline and tumour growth time-course was measured by BLI in a cohort of NRL-BM mice (n=5) in which liver tumours were induced by an i.v. injection of AAV8-TBG-Cre vector shortly after the first imaging time-point. Individual data points represent total photon flux (photons/s) measured for each animal at respective imaging time-points from a constant-area ROI placed over the chest in LivingImage 4.5 image analysis software. Mouse shown in panel c and d is labelled c/d. AR, autoradiography. **(c)** Example sequence of images acquired from an animal of the cohort described above prior to (0) and at 180, 210, and 240 days from tumour induction. **(d)** Example static [^18^F]TFB-PET-MRI image acquired from a NRL-BM animal 240 days after tumour induction. Tu, tumour; Th, thyroid; St, stomach; Bl, bladder. **(e)** Quantification of [^18^F]TFB-PET performed in NRL-BM (n=5 mice) or BM (n=4 mice), in which liver tumours had been induced by an i.v. injection of AAV8-TBG-Cre vector, or NRL-BM (n=4 mice) that had received an i.v. injection of AAV8-TBG-Empty vector. Each data point represents SUV_max_ measured for a liver tumour or a normal liver ROI using VivoQuant image analysis software (number of regions are 16, 6, 4, 14 respectively). Independent mice are represented with different colours. Images were acquired 70–90 minutes after an i.v. injection of [^18^F]TFB at approximately 500 MBq/kg. Ordinary, one-way, multiple comparisons ANOVA was performed to test statistical difference between the groups, and P values were calculated using Dunnett’s multiple comparisons test, evaluating all conditions versus the NRL-BM tumour, ** p < 0.01, *** p < 0.001. **(f)** Example autoradiography images of liver tumour sections collected from mice from the NRL-BM and BM cohorts that had been injected with equivalent (body-weight normalised and decay-corrected) doses of [^18^F]TFB 90 minutes before the animals were culled to collect tissue samples. **(g)** Example NIS/Slc5a5 RNAScope images of mNIS distribution in sections of liver tumours harvested at endpoint from NRL-BM and BM mice infected with AAV8-TBG-Cre vector. Scale bars represent 100 μm.

A critical advantage of developing an inducible multiscale imaging reporter system is to track the presence, expansion, and movement of cell populations over time. We therefore tested the utility of the R26^LSL – NRL^ reporter allele in the context of tumour formation in a conditional mouse model of hepatocellular carcinoma combining two gain-of-function Cre-inducible alleles *Ctnnb1*^*ex3/wt*^ and *R26*^*LSL − MYC/wt*^ (BM) [33, 39]. We induced liver tumours by intravenous injection of AAV8-TBG-Cre into NRL-BM and BM (littermate control) mice (Fig. 3a).

Using longitudinal bioluminescence imaging of Luc2 signal we visualised and localised oncogenesis over 12 months following induction (Fig. 3b, 3c), while both AAV8-TBG-Cre/BM and AAV8-TBG-empty/NRL-BM control mice showed no increase in signal over 56 weeks (Supplementary Fig. 5a). Tumours developed with varying burden and latency, and bioluminescence imaging facilitated the selection of animals for tomographic [^18^F]TFB PET-MR imaging, which permitted disease characterisation with the precise location of R26^NRL^ lesions co-registered with anatomical MR images (Fig. 3d). PET signal within the NRL-BM tumours was homogeneous and PET Quantification (maximum standard uptake value, SUV_max_) was clearly high in the reporter gene-expressing tumours unlike the background liver (Fig. 3e). PET signal was also greater than in lesions developed by littermate BM controls and untransformed liver tissue of NRL-BM animals infected with AAV8-TBG-empty control virus (Fig. 3e). [^18^F]TFB biodistribution (SUV_mean_) closely matched the PET imaging Quantification (Supplementary Fig. 5b), consistent with homogeneous tracer uptake. Homogenous distribution and high contrast with surrounding liver was further confirmed by autoradiography analysis (Fig. 3f) and RNAScope detection of NIS/Slc5a5 mRNA (Fig. 3g) in sections of tumours collected from NRL-BM and control BM mice. There was no background NIS/Slc5a5 expression in the livers from NRL-BM or control BM and NRL-BM/Empty mice (Supplementary Fig. 5c). Out of eight NRL-BM animals administered AAV8-TBG-Cre virus, four (50%) developed lesions with Luc2 bioluminescence and [^18^F]TFB PET signal, one (13%) developed lesions that did not produce measurable signal in either modality, two (25%) developed no macroscopic lesions within 12 months from induction. We culled one animal due to an unrelated health issue before tumours could appear. Together, these results show that combining the R26^LSL – NRL^ allele with conditional oncogenes can visualise tumour formation using multiple complementary imaging modalities.

### Triple modality reporter gene imaging of the R26^LSL – NRL/wt^ - Kras^LSL–G12D/wt^ - Trp53^fl/fl^ (NRL-KP) GEMM of lung carcinoma.

**Figure 4:**
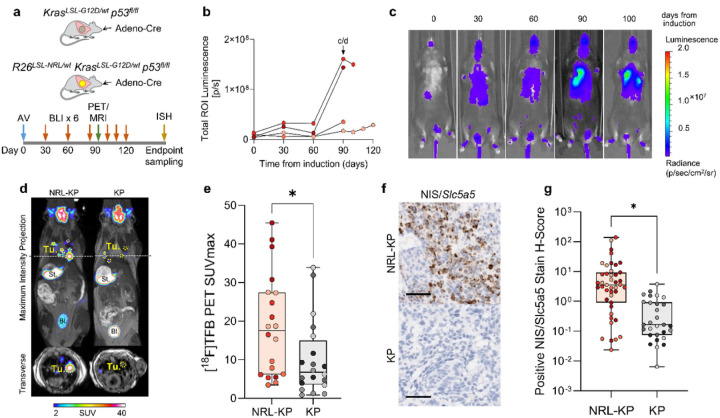
Multimodal imaging of tumour progression in the NRL-KP (*R26*^LSL-NRL/wt^ - *Kras*^LSL-G12D/wt^ - *Trp53*^fl/fl^) GEMM of lung cancer. **(a)** R26^LSL–NRL^ reporter mice were crossed with the gain-of-function *Kras*^LSL–G12D^ and the loss-of-function Trp53^fl/fl^ Cre- dependent alleles (KP) to generate a conditional mouse model of lung adenocarcinoma. Lung tumours were induced by intranasal inhalation of Ad5CMVCre into NRL-KP and KP (littermate control) mice before longitudinal bioluminescence imaging followed by PET-MRI and *ex vivo* Slc5a5 in situ hybridisation (ISH). **(b)** Tumour growth time-course measured by BLI in a cohort of NRL-KP mice (n=4) in which lung tumours were induced by intranasal inhalation of Ad5CMVCre vector shortly after the first imaging time-point. Individual data points represent total photon ux (p/s) measured from a constant-area ROI over the chest in LivingImage 4.5 software. Mouse shown in panel c and d is labelled c/d **(c)** Example sequence of images acquired from an animal (described in Fig. 4b) shortly prior to (‘0 days from induction’) and at 30, 60, 90 and 100 days from tumour induction. (d) Example static [^18^F]TFB PET-MRI image acquired from the NRL-KP animal in panel c 90 days from tumour induction. Tu, tumour; St, stomach; Bl, bladder. **(e)** Quantification of [^18^F]TFB-PET performed in the NRL-KP mice (n = 4 mice; n = 20 tumours; 5/mouse) or littermate KP animals (n = 4 mice; n = 20 tumours; 5/mouse) 90–100 days from tumour induction. Each data point represents SUV_max_ measured for a lung tumour ROI using VivoQuant image analysis suite. Images were acquired 70–90 minutes from the i.v. injection of [^18^F]TFB at 500 MBq/kg body weight. Independent mice are represented with different colours. Data were compared using an unpaired two-tailed t test, * p < 0.05. **(f)** Example Slc5a5 RNAScope images of mNIS/Slc5a5 distribution in sections of lung tumours harvested from NRL-KP mice or KP littermates. Scale bar represent 100 μm. **(g)** RNAScope detection of Slc5a5 mRNA in lung sections collected 90–100 days from tumour induction from NRL-KP (n = 4 mice; n = 43 tumours) or littermate KP (n = 4 mice; n = 29 tumours) animals. Each data point represents positive stain H-score calculated for a tumour ROI using HALO analysis suite. Data were compared using an unpaired two-tailed t test, * p < 0.05.

Having demonstrated the utility of the R26^LSL – NRL^ reporter allele in the context of liver cancer, we proceeded to test it on the background of a conditional mouse model of non-small cell lung adenocarcinoma by combining it a heterozygous conditional gain-of-function Kras^LSL−G12D^ allele and homozygous loss-of-function *Trp53*^fl/fl^ alleles (KP) (Fig. 4a). While tumour monitoring in the BM model, owing to the relatively large lesion size and their anatomical location, can be augmented by palpation, this is not possible in the case of autochthonous lung cancer models like the KP model. These models rely on monitoring disease symptoms, which is often inadequate since they may not become apparent until late stages. Furthermore, anatomical imaging of the lesions is complicated by breathing movements, the vicinity of the heart, and vascular and airway arbours.

All of the KP animals that received intranasal inhalation of Ad5CMVCre virus developed lung lesions, which we monitored with bioluminescence (BLI) over a period of 120 days from tumour induction (Fig. 4b, 4c). [^18^F]TFB PET imaging enabled the identification of individual lung lesions with high contrast over background (Fig. 4d). The average tumour SUV_max_ was significantly higher for NRL-KP mice in comparison to the littermate KP controls (18.1 ± 13.2 vs 10.0 ± 9.9; p = 0.026) (Fig. 4e). RNAScope detection of Slc5a5 mRNA showed higher positive stain H-scores in NRL-KP mice in comparison to KP littermate tumours with mean of 12.6 ± 27.5 vs. 0.61 ± 0.86, p = 0.02 in unpaired, two-tailed t-test (Fig. 4f, 4g). The expression pattern observed in RNAScope images of tumour tissue sections was variable, indicating intra- as well as inter-tumoural mNIS expression heterogeneity. Nevertheless, high specificity of the BLI signal in conjunction with high contrast tomographic [^18^F]TFB PET-MR imaging allowed positive identification and precise anatomical localisation of the NRL-KP lesions.

**Figure 5:**
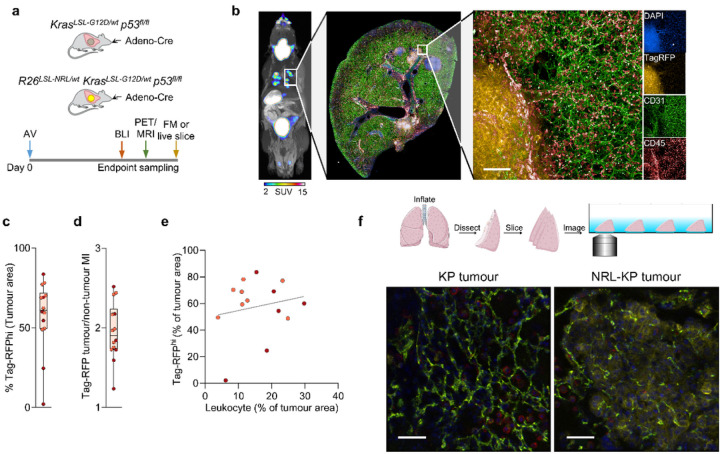
Fluorescence microscopy in the NRL-KP (*Kras*^LSL-G12D/wt^ *Trp*53^fl/fl^) GEMM of lung cancer. **(a)** R26^LSL–NRL^ reporter mice were crossed with the gain-of-function *Kras*^LSL–G12D^ and the loss-of-function *Trp53*^fl/fl^ Cre-dependent alleles (KP) to generate a conditional mouse model of lung adenocarcinoma. Lung tumours were induced by intranasal inhalation of Ad5CMVCre into NRL-KP and KP (littermate control) mice before bioluminescence (BLI) imaging, [^18^F]TFB PET-MRI followed by *ex vivo* fluorescence microscopy (FM) imaging performed on fixed or live sections of tumour containing lung tissue. **(b)** [^18^F]TFB PET-MRI followed by ex vivo fluorescence microscopy (FM) imaging performed on fixed sections of tumours harvested from a cohort of NRL-KP animals stained with anti-CD31 (green), anti-CD45 (red) and DAPI (blue). Tumours cells are visible due to TagRFP (yellow). Scale bar is 100 μm. **(c)** Percentage of tumour area displaying high RFP signal estimated in a sample (n=2 mice, 14 tumours) of tumours harboured by NRL-KP animals **(d)** Ratios (tumour: normal tissue) of mean RFP signal intensity of the sample of tumours (n=2 mice, 14 tumours) **(e)** High TagRFP expression versus leukocyte infiltration (described as % tumour area; (n=2 mice, 14 tumours; independent mice have different colours) **(f)** Live Precision Cut Lung Slices (PCLS). Single frames from movies (Supplementary Video 1) of PCLS harvested from KP or NRL-KP littermates. Scale bar is 50μm.

To determine whether the tumour cells detectable by bioluminescence and [^18^F]TFB-PET imaging could also be detected by flfluorescence microscopy, we induced tumours in two NRL-KP mice 12–16 weeks before imaging with bioluminescence and [^18^F]TFB PET-MR. Precision cut lung slices (PCLS) were then produced prior to flfluorescence microscopy (Fig. 5a and 5b). These experiments demonstrated that 57.8 ± 21.9% of the NRL-KP tumour area comprised cells exhibiting high levels of TagRFP fluorescence across 14 tumours from two NRL-KP mice (Fig. 5c). TagRFP fluorescence signal was 1.95 ± 0.36 times greater in the tumour compared to non-tumour lung tissue (Fig. 5d). Using CD45-AF647 co-staining, the abundance of TagRFP high cells did not appear to correlate with leukocyte infiltration of the NRL-KP tumours (simple linear regression coefficient r^2^ = 0.035, p = 0.052; Fig. 5e), suggesting that leukocytes did not preferentially infiltrate highly labelled tumours. To image immune cell dynamics we stained live PCLS with fluorescently tagged antibodies targeting endothelial cells (anti-CD31 AF647) and leukocytes (anti-CD45 AF488) from one KP tumour and one NRL-KP tumour (Fig. 5f). These experiments demonstrated the feasibility of imaging cell-cell interactions between endothelial and immune cells within the milieu of NRL-KP tumour cells, which could be clearly identified by their TagRFP fluorescence (Fig. 5f and Supplementary Video 1). This is particularly advantageous in tumour types that do not express a specific surface antigen suitable for antibody tagging as the fluorescent reporter could be used for reliable tumour cell segmentation in imaging analysis. Together, these data show that the R26^LSL–NRL^ reporter allele enables tracking of marked cells during oncogenesis at the whole animal level, specific tumours can be localised and quantified using PET imaging and these same tumour cells can be imaged interacting with their local tumour microenvironment.

### Multiscale reporter gene imaging of the R26^LSL – NRL/wt^ - Kras^LSL−G12D/wt^ - Trp53^fl/fl^ (NRL-KP) GEMM of lung carcinoma.

**Figure 6:**
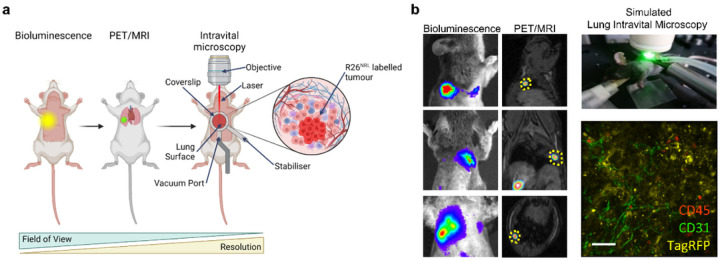
Multiscale tumour imaging in the NRL-KP (*Kras*^LSL-G12D/wt^ Trp53^fl/fl^) GEMM of lung cancer. **(a)** Experimental schematic showing tumour identification by bioluminescence imaging, accurate localisation by PET-MRI guiding simulated Lung Intravital Microscopy (S-LIVM) to discrete NRL-KP lung tumours. **(b)** Identification of skin landmarks by bioluminescence imaging allowing the best route of approach to the tumour. Fine anatomical localisation of the tumour using PET-MRI. A single static PET image was acquired between 70–90 minutes from the i.v. injection of [^18^F]TFB at 500 MBq/kg. S-IVM experimental set-up. Single frame from an S-LIVM movie (Supplementary Video 2). Anti-CD31 (green), anti-CD45 (red) and TagRFP (yellow). Scale bar is 50 μm.

To investigate whether BLI and PET could stratify mice most suitable for intra-vital microscopy by precise identification and localisation of reporter signal-positive lung lesions, we induced a small cohort of animals (n = 4) with a low-titre dose of Ad5CMVcre (5 × 10^6^ PFU) to produce tumours dispersed throughout the lung volume, while limiting overall tumour burden (Fig. 6a). Based on serial BLI and [^18^F]TFB PET-MR imaging, we identified a cluster of lesions adjacent to the rib cage in one of these mice. To mark the best route of access to this tumour we then placed marker pen dots approx. 3 mm in diameter on the animal’s skin during a bioluminescence imaging session. These cues guided surgical incision to expose the correct inter-costal space for an imaging window to allow simulated Lung Intravital Microscopy (S-LIVM) of immune cells, capillaries and tumour cells in the mechanically ventilated lung of a humanely killed mouse (Fig. 6b). These studies demonstrated the feasibility of triple reporter gene guided tumour cell imaging from whole animals to microscopy and the power of stratifying the cohort by those most likely to have an ‘imageable’ tumour location.

## Discussion

This study aimed to address an important gap in the arsenal of tools for reporter gene imaging, specifically, the ability in the scope of one reporter system, to flexibly switch scales from whole-animal, to cellular or subcellular imaging, exploiting the fundamental advantages of multi-modal imaging. We developed a novel multimodal germ-line transgenic reporter cassette encoding a self-cleaving mNIS-TagRFP-P2A-Luc2 sequence, expressed from the CMV early enhancer/chicken β-actin (CAG) promoter and featuring a Cre-dependent conditional stop element (lox-STOP-lox). Through crossing the R26^LSL – NRL^ transgenic cassette with ubiquitously expressing Cre recombinase lines, including constitutively active CMV-Cre [31] or the tamoxifen-inducible Cre isoform R26^Cre − ERT2^ [32], we demonstrated the inducibility of functional multi-modal imaging. Bioluminescence and [^18^F]TFB PET signals were detected in every organ analysed, indicating high-level and stable R26^NRL^ expression across various tissue types, facilitating multi-modality imaging.

The R26^LSL – NRL^ multimodal reporter gene system enabled precise visualisation and monitoring of oncogenesis in multiple spontaneously forming tumours originating from different tissues which are challenging to detect with palpation or other imaging modalities due to their heterogeneous latency and deep tissue location [6]. While BLI facilitated rapid screening, PET imaging allowed for tomographic tumour detection with accurate Quantification, enabling visualisation of individual lesions in three-dimensional space. Integration of whole-body mapping to intra-vital microscopy allowed tumour triangulation, facilitating in-situ microscopic imaging of dynamic interactions between tumour cells and their microenvironment. Through multi-scale imaging, specific tumours were identified and characterised, growth was monitored, and interactions between tumour and immune cells were visualised. Notably, the observed immune infiltration did not correlate with the level of TagRFP reporter gene expression, suggesting that the immune system did not preferentially respond to tagged tumours.

This conditional triple reporter system offers several advantages over other potential approaches. For example, while somatic gene transfer of both Cre and reporter gene sequences on the background of a conditional GEMM is resource-efficient, it may be limited due to lack vector tropism [40]. There are also payload considerations, with a ceiling at approximately 10 kbp for lentiviral vector, limiting the total number of reporter genes that are deliverable somatically [41]. Combining different transgenic alleles in a single composite animal through cross-breeding although straightforward in principle, requires prolonged and costly breeding. Moreover, as the R26^LSL – NRL^ allele and several transgenic alleles related to modelling human disease occupy a small set of permissive loci such as Rosa26 [8–10, 28], there is a limit on the number of reporter transgenic alleles that can be combined by simple cross-breeding. While alternative loci and new transgenic technologies, like CRISPR/Cas9 may alleviate this, establishing and characterising new reporter strains is time consuming and costly.

Multimodal imaging of labelled cells with a triple reporter mouse is attractive as it facilitates sensitive tumour cell imaging at different scales. Rather than fuse all constituent reporter transgenes together and risk compromising performance, we chose to employ a 2A sequence to ensure equimolar expression and gene function of all reporter transgenes. NIS function is minimally affected by the fusion of a fluorescent protein to its C-terminal [17]. We employed that strategy here, as in addition to conferring a fluorescent readout to labelled cells, it enables visualisation of the intracellular location of NIS protein, which needs to be transmembrane to be functional.

Switching of the Cre-dependent reporter cassette generally marked the majority of the lesions with reporter transgene expression, however there was a subset of tumours that did not produce a detectable increase of signal. This may be an effect of incomplete recombination at induction, wherein successful Cre-recombination occurs at the proto-oncogenic Cre-loxP loci but not at the R26^LSL – NRL^ locus. Further, the genomic location of the NRL reporter cassette within the Rosa26 locus could limit the broad applicability of our approach. The Rosa26 locus as gained popularity in genome transgenesis due to its high targeting efficiency. Consequently, the R26^LSL – NRL^ triple reporter may be restricted to GEMMs possessing one or fewer R26 transgenic elements.

The switchable expression and targeted imaging of various tissues in the R26^LSL – NRL^ mouse, enables multiscale cell tracking in a range of research domains including developmental biology, neuroscience, immunology, oncology and regenerative medicine. Labelling immune cells opens up the possibility of crossing the R26^LSL – NRL^ mouse immune specific lines like Ly6G-Cre providing opportunity to image the role neutrophils in the pathogenesis of inflammatory arthritis and cancer [42, 43]. In oncology, this reporter system could be used to track a wide variety of cell types and tumour cells during evolution from oncogenesis to metastatic seeding, as well as for monitoring therapeutic interventions and evaluating treatment efficacy in the whole-body and cellular scale in real-time [26]. The versatility of the R26^LSL – NRL^ model allows for the utilisation of both optical and radionuclide approaches, addressing the limitations associated with these as single modalities. This versatility allows complementary improvements in quantitative accuracy, tomography, high throughput imaging, as well as detailed imaging of cellular interactions. For example, when coupled with the oncogenic Kras/p53 null lung cancer model, which is widely used for non-small cell lung cancer research despite its inherent inter-tumour heterogeneity, the R26^LSL – NRL^ system enables the imaging and monitoring of discrete tumours [44, 45]. We have recently demonstrated rapid assessment of tumour response using mNIS therefore this reporter mouse strain offers the opportunity to characterise individual tumour responses, significantly enhancing our understanding of regional tumour vulnerabilities in lung cancer [26]. Given the prevalent use of the Cre-loxP system in preclinical research, this reporter system facilitates correlative multiscale imaging across a broad range of applications, serving as a new tool for investigating gene expression, cell behaviour, and whole-body disease processes *in vivo*. The R26^LSLNRL^ multimodal reporter gene system represents a robust and versatile platform for precise in vivo imaging across multiple scales, addressing key limitations of existing approaches and enabling comprehensive insights into complex biological processes and disease dynamics.

## Figures and Tables

**Table 1.** Summary of gating used in flow cytometry experiments

Cell Type	Gating
eosinophils	CD19- CD3- NKp46- CD11b+ SiglecF+ Ly6G-
neutrophils	CD19- CD3- NKp46- CD11b+ SiglecF- Ly6G+
dendritic cells	CD19- CD3- CD11c+
natural killer cells	CD19- CD3- NKp46+ CD11 blo/mid
B cells	CD19+ CD3-
CD8 T cells	CD19- CD3+ CD4- CD8+
CD4 T cells	CD19- CD3+ CD4+ CD8-
γδ T cells	CD19- CD3+ CD4+ CD8- TCRγδ+

## Data Availability

PET/MRI and microscopy imaging data, histology data, and data tables are available on request.
